# Can Psychological Readiness Predict Successful Return to Sports After Patellar Stabilization Surgery in Adolescent Patients? A Retrospective Cohort Study

**DOI:** 10.1177/03635465261487282

**Published:** 2026-10-09

**Authors:** Caroline L. Kim, Ryan W. Turlip, Monika Thothadri, Lauren H. Redler, Elliot Greenberg, Brendan A. Williams

**Affiliations:** * Perelman School of Medicine, University of Pennsylvania, Philadelphia, Pennsylvania, USA; † Orthopedic Center, Children’s Hospital of Philadelphia, Philadelphia, Pennsylvania, USA; ‡ Department of Orthopaedic Surgery, Columbia University Medical Center, New York, New York, USA; Investigation performed at the Orthopedic Center, Children’s Hospital of Philadelphia, Philadelphia, Pennsylvania, USA

**Keywords:** patellofemoral instability, psychological factors, return to sports, patient-reported outcome questionnaires

## Abstract

**Background:**

While many metrics are used to subjectively assess a patient’s knee function with patellofemoral instability (PFI), psychological readiness to return to sport (RTS) after patellar stabilization surgery is not well established in this population. The Anterior Cruciate Ligament Return to Sport After Injury (ACL-RSI) scale assesses psychological readiness to RTS after anterior cruciate ligament reconstruction and may be similarly beneficial in understanding this domain in those with PFI.

**Hypothesis:**

Higher ACL-RSI scores in patients with PFI are associated with an increased likelihood of RTS after patellar stabilization surgery.

**Study Design:**

Cohort study; Level of evidence, 3.

**Methods:**

A retrospective review was conducted of adolescent patients (10-19 years old) treated surgically for PFI (medial patellofemoral ligament reconstruction [MPFLR] with or without tibial tubercle osteotomy) at a single institution from January 2020 to October 2024. Patients actively participating in sports at their first orthopaedic visit who completed the ACL-RSI during postoperative rehabilitation were included. Patient demographics, injury characteristics, surgical procedure, ACL-RSI scores, and RTS status were recorded. Descriptive statistics and bivariate analysis were performed. Based on the euclidean distance, the optimal ACL-RSI cutoff score for predicting successful RTS was determined, and diagnostic accuracy was evaluated with a receiver operating curve. A multivariate logistic regression, adjusted for sex, was used to identify predictors of RTS.

**Results:**

Study criteria identified 97 patients with a median (IQR) age of 15.6 years (14.2-16.4). Seventy underwent isolated MPFLR and 27 underwent MPFLR with tibial tubercle osteotomy. Successful RTS was achieved in 78.3% (n = 76). RTS was associated with higher ACL-RSI scores (*P* = .012), isolated MPFLR (*P* = .011), and male sex (*P* = .03). An ACL-RSI score ≥59.2 was identified as being indicative of successful RTS (area under the curve, 0.70; 95% CI, 0.56-0.83). While procedure type and ACL-RSI scores were both associated with RTS in univariate analysis, the multivariable analysis adjusting for sex revealed that the ACL-RSI cutoff remained the only significant predictor of RTS (odds ratio, 3.56; 95% CI, 1.10-11.51; *P* = .033).

**Conclusion:**

ACL-RSI scores ≥59.2 are associated with an increased likelihood of successful RTS after surgical treatment for PFI in adolescents. Patient demographics and the studied injury characteristics do not appear to influence the likelihood of successful RTS.

Patellofemoral instability (PFI) commonly affects young athletes.^10,13,15^ After a first-time patellar dislocation, up to 60% of patients have recurrent feelings of instability or dislocation events, which can be disconcerting and limit willingness to participate in some activities, including sports.^5,14^ Patients with recurrent PFI often undergo surgical stabilization, with up to 85% successfully returning to sports.^10,15^ Fear of reinjury, however, is commonly cited by patients with PFI as a barrier to returning to sports after medial patellofemoral ligament reconstruction (MPFLR), even after functional goals are met.^17^ While many metrics are available to objectively assess postoperative physical function, psychological readiness is not well established in this population.

The Anterior Cruciate Ligament Return to Sport After Injury (ACL-RSI) scale assesses confidence in knee performance, fear of reinjury, and emotional responses to returning to sport.^23^ Scores between 56 and 65 have been established as being predictive of successful return to sport (RTS) after anterior cruciate ligament reconstruction (ACLR).^1,18^ Although the ACL-RSI was developed primarily for use within the ACLR population, the questions within the ACL-RSI are phrased in general terms referring to the knee rather than specifically to the anterior cruciate ligament. Hence, the scale’s lack of specificity to the anterior cruciate ligament may allow for its application to populations with other knee pathology, potentially serving as a useful tool to inform rehabilitation and RTS decision-making in these settings.^22^ Assessment of psychological readiness with the ACL-RSI may be particularly beneficial for patients undergoing patellar stabilization surgery.

Given the dearth of tools to quantify the psychological aspect of recovery in patients undergoing patellar stabilization surgery, we sought to evaluate factors associated with successful RTS, including the predictive capacity of the ACL-RSI in this population. We hypothesized that higher ACL-RSI scores in patients with PFI are associated with an increased likelihood of RTS after patellar stabilization surgery.

## Methods

After ethical approval for this study was obtained from the Children’s Hospital of Philadelphia (IRB 14-010853), we performed a retrospective review of adolescent patients (10-19 years old) treated surgically for PFI with MPFLR, with or without tibial tubercle osteotomy (TTO), at a single institution from January 2020 to October 2024. Patients actively participating in sports at the first orthopaedic visit who completed the ACL-RSI during postoperative rehabilitation were included. Patients with a history of same-knee surgery or patients undergoing less common concomitant bony procedures (eg, trochleoplasty or distal femoral osteotomy) were excluded.

Patient-specific variables included age, sex, race, and body mass index. Injury characteristics were recorded, including the frequency of instability events and the type (subluxation or dislocation). Postoperative symptomatic instability, ACL-RSI short form scores, and RTS status after patellar stabilization surgery were determined. Successful RTS was defined by the patient returning to the same sport and/or the same level of athletics in which they participated before surgery.

The ACL-RSI short form is a validated 6-item abbreviated version of the original ACL-RSI designed to assess psychological readiness to RTS after ACLR^22^ (Figure 1). ACL-RSI short form questionnaires were completed as part of institutional standard of care during postoperative physical therapy visits. Scores were categorically grouped into common postoperative follow-up timepoints: 6 weeks (≤6 weeks), 3 months (>6-12 weeks), 6 months (>12 weeks-6 months), 9 months (>6-9 months), and >9 months.

**Figure 1.**
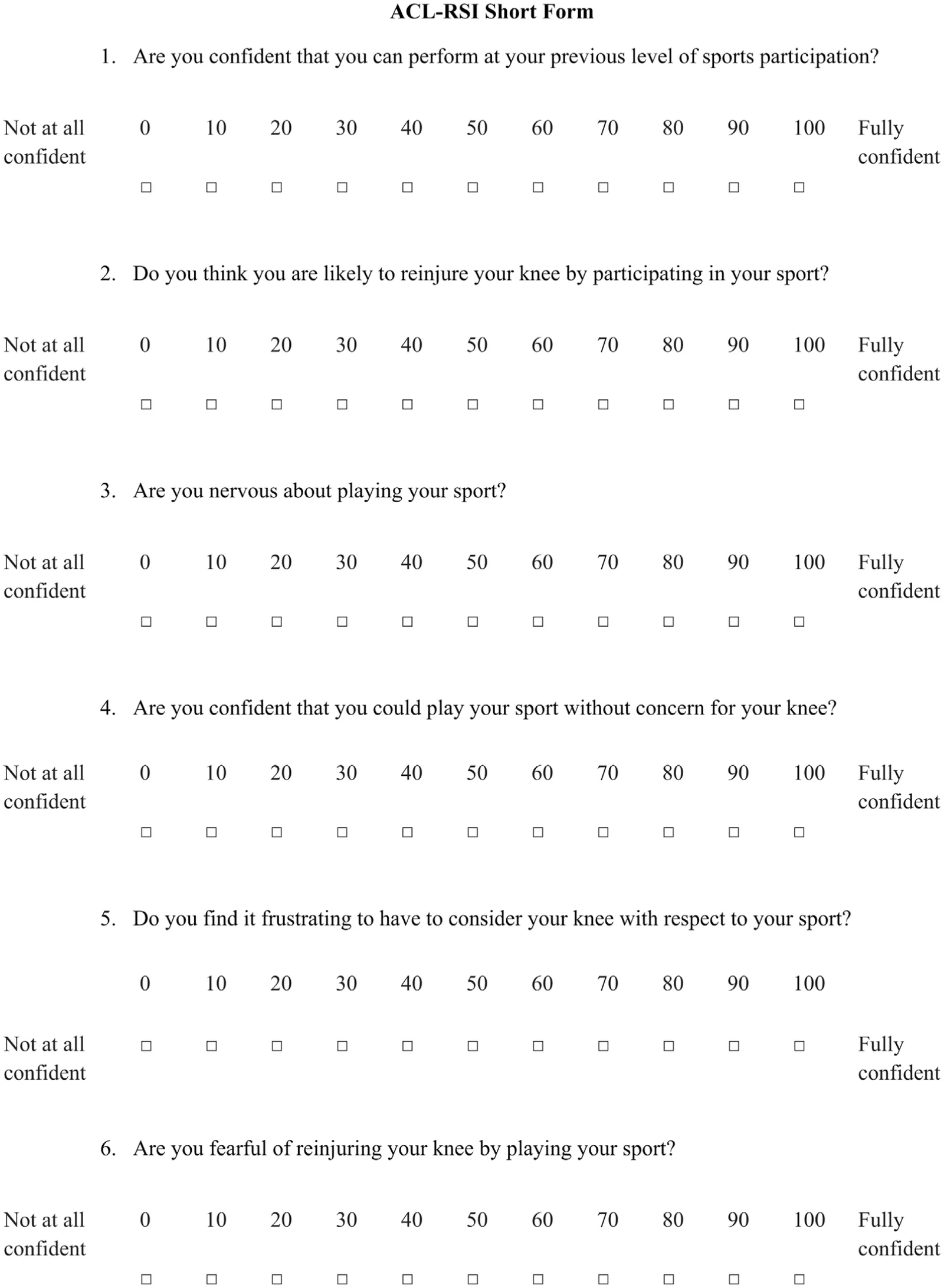
ACL-RSI short form survey adapted from Webster and Feller.^22^ ACL-RSI, Anterior Cruciate Ligament Return to Sport After Injury.

The Shapiro-Wilk test was used to determine the normality of distributions. Continuous variables were analyzed by the Mann-Whitney *U* test, and categorical variables were compared by Pearson chi-square or Fisher exact test, as appropriate. To evaluate changes in ACL-RSI scores over time and account for repeated measurements within participants, a mixed effects model was used. Timepoint and procedure type were included as fixed effects, with the participant modeled as a random effect. Timepoint was treated as a categorical variable, with >9 months used as a reference category. An interaction between timepoint and procedure type was included to evaluate whether temporal changes in ACL-RSI score differed by procedure type. The optimal ACL-RSI cutoff score for predicting successful RTS was determined by euclidean distance, and diagnostic accuracy (area under the curve) was evaluated with a receiver operating characteristic curve. Kaplan-Meier analysis was applied to determine the time to achieve the cutoff (59.2), and the median estimated time was calculated for the overall cohort and across the procedure groups. A multivariable logistic regression model was fitted using significant variables to determine the predictors of RTS and was adjusted for sex. Sex-specific subanalyses were additionally performed to evaluate the association between RTS and procedure type within each sex. All analyses were performed in R (Version 4.5.1) and SAS software (Version 9.4). Statistical significance was set at *P* < .05.

## Results

Study criteria identified 97 patients with a median age of 15.6 years (IQR, 14.2-16.5): 70 patients (72.1%) underwent isolated MPFLR while 27 (27.8%) underwent MPFLR + TTO. Patients undergoing MPFLR + TTO included a higher proportion of female patients (74.1% vs 47.1%; *P* = .02) and a higher proportion of patients who experienced recurrent patellar instability preoperatively (96.3% vs 74.3%; *P* = .02) as compared with patients undergoing isolated MPFLR. On preoperative imaging, patients undergoing MPFLR + TTO had a greater tibial tubercle–trochlear groove distance than the isolated MPFLR group (16.0 vs 21.0 mm; *P* < .001). No other demographic differences were observed between the surgical groups (*P* > .05; Table 1).

**Table 1:** Descriptive Analysis of Demographic, Clinical, and Surgical Factors Between Surgical Groups*^*a*^*

Characteristic	Overall (N = 97)	Isolated MPFLR (n = 70)	MPFLR + TTO (n = 27)	*P* Value*^*b*^*
Sex				**.023**
Female	53 (55)	33 (47)	20 (74)	
Male	44 (45)	37 (53)	7 (26)	
Age, y	15.6 (14.2-16.4)	15.3 (14.1-16.4)	16.0 (14.7-17.1)	.07
Body mass index	22.1 (19.4-25.0)	22.0 (19.5-25.0)	22.4 (19.1-25.4)	.95
Race				.520
White	70 (72)	52 (74)	18 (67)	
Black	15 (16)	9 (13)	6 (22)	
Other	12 (12)	9 (13)	3 (11)	
Injury type				.755
Contact	15 (15)	10 (14)	5 (18)	
Noncontact	82 (84)	60 (85)	22 (81)	
Instability type				**.026**
Dislocation	87 (90)	66 (94)	21 (78)	
Subluxation	10 (10)	4 (6)	6 (22)	
Recurrent				
No	19 (20)	18 (26)	1 (4)	**.0198**
Yes	78 (80)	52 (74)	26 (96)	
TT-TG, mm	17.0 (13.5-19.4)	16.0 (12.0-18.0)	21.0 (19.0-24.0)	**<.001**
CDI	1.27 (1.10-1.40)	1.25 (1.10-1.39)	1.35 (1.10-1.45)	.165

a Data are presented as n (%) or median (IQR). CDI, Caton-Deschamps index; MPFLR, medial patellofemoral ligament reconstruction; TTO, tibial tubercle osteotomy; TT-TG, tibial tubercle–trochlear groove.

b Shapiro-Wilk, Mann-Whitney *U*, Pearson chi-square, Fisher exact. Bold indicates *P* < .05.

Overall, 76 patients (78.3%) successfully returned to sports by their final follow-up visit. RTS was associated with higher overall ACL-RSI scores (median, 65.8 vs 53.3; *P* = .012). Patients undergoing isolated MPFLR also returned to sports at a higher proportion than patients undergoing MPFLR + TTO (85.7% vs 59.2%; *P* = .011), and male patients returned to sports at a higher proportion than female patients (88.6% vs 69.8%; *P* = .028). No other demographic differences affected RTS rates (*P* > .05; Table 2). A mixed effects model revealed a significant effect of time on ACL-RSI (*P* < .0001). When compared with scores >9 months, ACL-RSI scores were significantly lower at 6 weeks (β = −33.2; *P* < .0001) and 3 months (β = −20.6; *P* < .01). There was no significant difference in the ACL-RSI scores between the procedure types across timepoints (*P* > .05; Figure 2). Of the patients who returned to sports, the overall median time to achieve an ACL-RSI score ≥59.2 was 8.93 months (95% CI, 7.35-9.92). In patients undergoing isolated MPFLR, the median time to achieve the cutoff score was 8.44 months as compared with 9.92 months in patients undergoing MPFLR + TTO.

**Table 2:** Descriptive Analysis of Demographic, Clinical, and Surgical Factors Associated With Successful Return to Sports*^*a*^*

Characteristic	Overall (N = 97)	Return to Sports (n = 76)	No Return (n = 21)	*P* Value*^*b*^*
Sex				**.028**
Female	53 (54)	37 (49)	16 (76)	
Male	44 (45)	39 (51)	5 (24)	
Age, y	15.6 (14.2-16.4)	15.5 (14.2-16.3)	15.8 (14.2-17.0)	.3952
Body mass index	22.1 (19.4-25.0)	22.2 (19.6-24.8)	21.3 (19.2-26.5)	.9826
Race				.3825
Black	15 (16)	10 (13)	5 (24)	
Other	12 (12)	9 (12)	3 (14)	
White	70 (72)	57 (75)	13 (62)	
Injury type				.5119
Contact	15 (16)	13 (17)	2 (10)	
Noncontact	82 (84)	63 (83)	19 (90)	
Instability type				.4473
Dislocation	87 (90)	69 (91)	18 (86)	
Subluxation	10 (10)	7 (9)	3 (14)	
Recurrent instability				
Yes	78 (80)	58 (76)	20 (95)	.0647
No	19 (20)	18 (24)	1 (5)	
Procedure type				**.0113**
Isolated MPFL	70 (72)	60 (79)	10 (48)	
MPFL + TTO	27 (28)	16 (21)	12 (52)	
ACL-RSI score	65.0 (50.0-85.0)	65.8 (51.7-87.5)	53.3 (38.3-66.7)	**.0119**

a Data are presented as n (%) or median (IQR). ACL-RSI, Anterior Cruciate Ligament Return to Sport After Injury; MPFLR, medial patellofemoral ligament reconstruction; TTO, tibial tubercle osteotomy.

b Shapiro-Wilk, Mann-Whitney *U*, Pearson chi-square, Fisher exact. Bold indicates *P* < .05.

**Figure 2.**
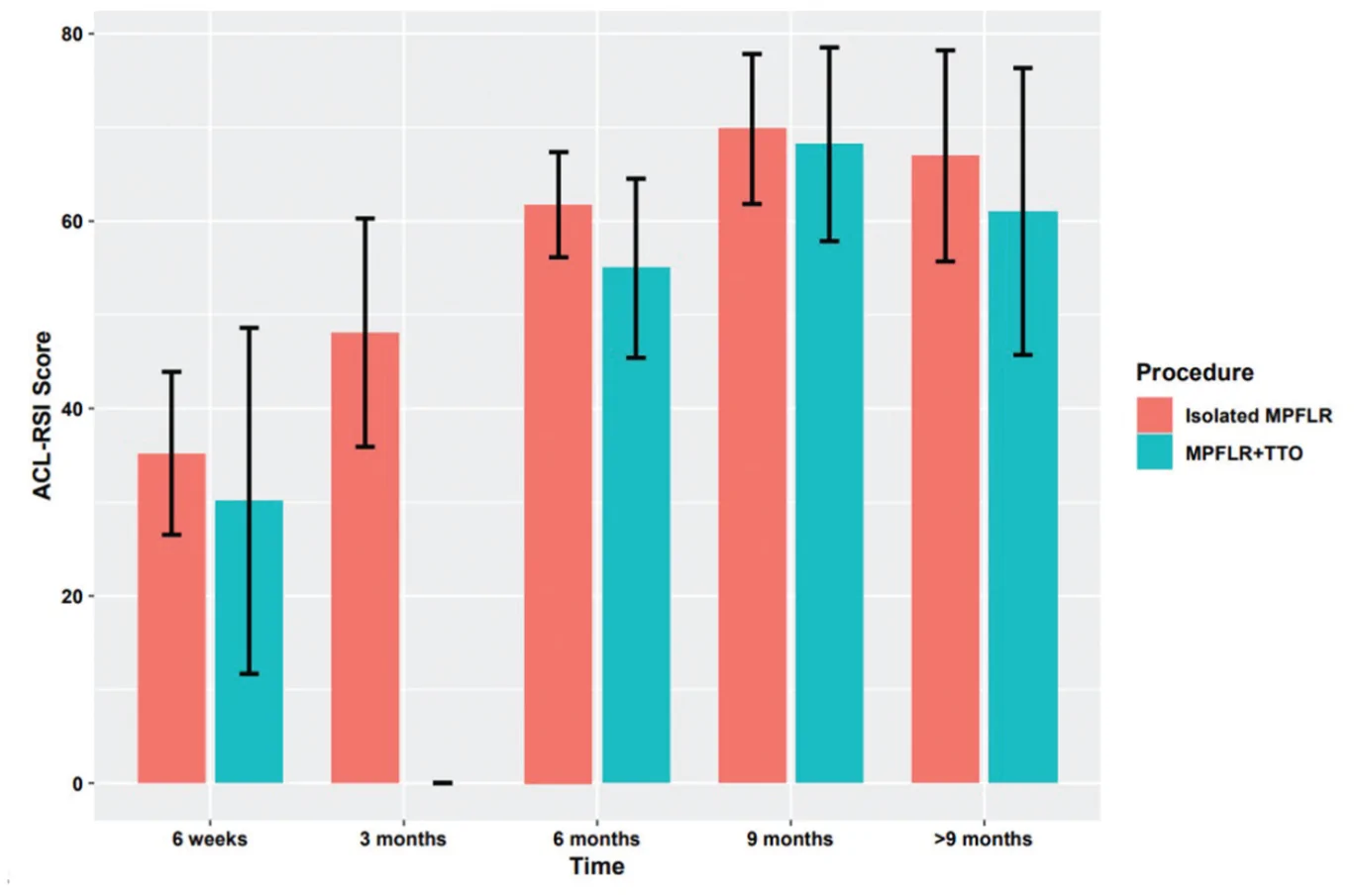
ACL-RSI scores by procedure across timepoints. ACL-RSI scores at 6 weeks (n = 27), 3 months (n = 11), 6 months (n = 74), 9 months (n = 42), and >9 months (n = 20) between patients undergoing isolated MPFLR and MPFLR + TTO. Differences in ACL-RSI scores across timepoints was overall significant (*P* < .0001). Post hoc group comparisons demonstrated significantly lower scores at 6 weeks (β = −33.2; *P* < .0001) and 3 months (β = −20.6; *P* < .01) as compared with scores >9 months. Data are presented as median (IQR). ACL-RSI, Anterior Cruciate Ligament Return to Sport After Injury; MPFLR, medial patellofemoral ligament reconstruction; TTO, tibial tubercle osteotomy.

According to the receiver operating characteristic curve, an ACL-RSI score ≥59.2 was identified as being indicative of successful RTS (area under the curve, 0.70; 95% CI, 0.56-0.83). At the optimal cutoff point, the ACL-RSI score demonstrated a sensitivity of 63%, specificity of 66%, and overall accuracy of 64% (Figure 3). A multivariable logistic regression model was fitted by the significant independent variables listed in Table 2. The model after adjusting for the sex revealed that the ACL-RSI cutoff remained the only statistically significant predictor associated with higher odds of RTS (odds ratio, 3.56; 95% CI, 1.10-11.51; *P* = .0333), whereas procedure type and sex were not associated with RTS (*P* > .05; Table 3).

**Figure 3.**
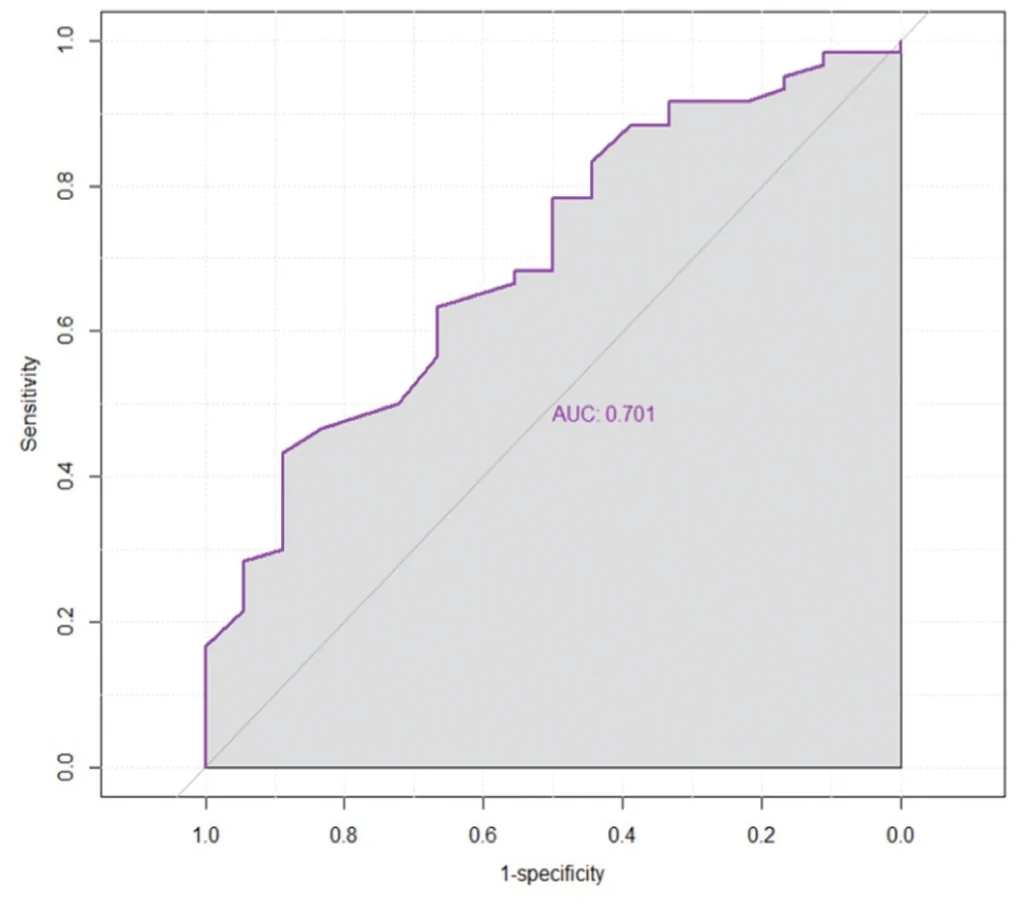
Receiver operating characteristic curve for ACL-RSI score predicting successful return to sport after patellofemoral instability surgery. The curve illustrates the discriminative ability of the ACL-RSI score in predicting successful return to sport. The area under the curve (AUC) was 0.70, indicating acceptable predictive performance. At the optimal cutoff point, the ACL-RSI score demonstrated a sensitivity of 63%, specificity of 66%, and overall accuracy of 64%. The diagonal line represents the line of no discrimination (AUC = 0.5). ACL-RSI, Anterior Cruciate Ligament Return to Sport After Injury.

**Table 3:** Multivariable Logistic Regression Evaluating Factors Associated With Return to Sports*^*a*^*

Factor Predicting Return to Sports	Odds Ratio (95% CI)	*P* Value
ACL-RSI cutoff scores ≥59.2 vs <59.2	3.56 (1.10-11.51)	**.0333**
Isolated MPFLR vs MPFLR + TTO	3.04 (0.88-10.47)	.0772
Female vs male	0.47 (0.13-1.68)	.2517

a ACL-RSI, Anterior Cruciate Ligament Return to Sport After Injury; MPFLR, medial patellofemoral ligament reconstruction; TTO, tibial tubercle osteotomy. Bold indicates *P* < .05.

In the sex-stratified subanalysis evaluating for RTS and procedure type (Table 4), we observed a statistically significant association between RTS and procedure type (with or without TTO) among females (*P* = .0045) but not among males (*P* = .5743).

**Table 4:** Return to Sports Stratified by Sex Subanalysis*^*a*^*

	Overall	Return to sports	No Return	*P* Value*^*b*^*
Return to sports for males	44	39	5	
Procedure type				.5743
Isolated MPFL	37 (84.09)	32 (82.05)	5 (100)	
MPFL + TTO	7 (15.91)	7 (17.95)	00 (0)	
Return to sports for females	53	37	16	
Procedure type				**.0045**
Isolated MPFL	33 (62.26)	28 (75.68)	5 (31.25)	
MPFL + TTO	20 (37.74)	9 (24.32)	11 (68.75)	

a Data are presented as n (%). ACL-RSI, Anterior Cruciate Ligament Return to Sport After Injury; MPFLR, medial patellofemoral ligament reconstruction; TTO, tibial tubercle osteotomy.

b Fisher exact test. Bold indicates *P* < .05.

## Discussion

The purpose of this study was to identify factors associated with successful RTS in adolescent patients undergoing patellar stabilization surgery with a focus on psychological readiness. We sought to evaluate the predictive capacity of the ACL-RSI generalized to the PFI population. Multivariable logistic regression identified ACL-RSI scores ≥59.2 as being associated with an increased likelihood of successful RTS. Patient demographic features and other injury characteristics did not appear to influence the likelihood of successful RTS.

Patients undergoing patellar stabilization surgery have high rates of RTS.^3,10,20^ MPFLR has emerged as one of the primary surgical treatments for treating PFI, with prior studies demonstrating good outcomes and low redislocation rates.^7,16,19^ Manjunath et al^10^ identified an 85.1% RTS rate across a systematic review of 1278 patients (mean age, 22.0 years) undergoing MPFLR. Migliorini et al^12^ reached similar conclusions among a review of 477 pediatric patients (mean age, 14.6 years) with 87% successfully returning to the same level of activities as before surgery. Consistent with this existing literature, our study identified that 85.7% of adolescent patients undergoing isolated MPFLR were able to RTS, supporting isolated MPFLR as a viable surgical procedure to help young athletes with PFI successfully return to high levels of activity. Further investigation into the specific level and intensity of sport participation upon return would enhance our understanding of athletic outcomes after patellar stabilization surgery and help optimize preoperative patient counseling and rehabilitation strategies.

TTO can be performed alongside MPFLR in patients with more severe anatomic risk factors, including lateralized extensor mechanisms and patella alta, to reduce the likelihood of recurrent instability.^21^ The impact of concomitant TTO performed alongside MPFLR on RTS remains unclear. Kew et al^8^ reported that only 66% of patients undergoing MPFLR + TTO successfully returned to sports as opposed to 85% of patients undergoing isolated MPFLR. In their systematic review, Li et al^9^ revealed that young adult patients undergoing MPFLR and MPFLR + TTO returned to preinjury-level sports at similar rates (67.6% vs 73.0%). Our study found that significantly fewer patients returned to sports who underwent MPFLR + TTO (59.2%) as compared with isolated MPFLR (85.7%; *P* = .003). This comparison, however, is limited given the unbalanced procedural cohorts and inherent selection bias related to demographic and anatomic differences among patients indicated for TTO. Within our cohort, the MPFLR + TTO group had a higher proportion of female patients (74.1% vs 47.1%; *P* = .02), patients with recurrent patellar instability (96.3% vs 74.3%; *P* = .02), and patients with a greater tibial tubercle–trochlear groove distance (16.0 vs 21.0 mm; *P* < .001). These findings suggest that patients undergoing MPFLR + TTO likely had more severe anatomic and demographic risk factors for patellar instability at baseline. Therefore, the lower RTS rate among patients undergoing MPFLR + TTO should not be attributed solely to the addition of TTO but may reflect the more severe risk factors that warranted a concomitant TTO. As a result, we feel that direct comparisons of RTS rates between these surgical procedures are not appropriate within this study cohort. This rate is also notably lower than the 66% and 73% RTS rates reported by Kew et al and Li et al, respectively. Several factors may contribute to this discrepancy. Most notably, our cohort was substantially younger (median age, 15.56 years) than the populations studied by Kew et al and Li et al (mean ages, 24 and 26 years). This younger age may reflect earlier symptom onset, more severe anatomic risk factors, or higher level of athletic participation, though not directly compared in this study. Younger athletes may also face unique challenges in returning to sports, including higher preinjury activity demands. Further studies in larger cohorts are warranted to better characterize expected outcomes and to identify factors contributing to lower RTS rates among patients undergoing MPFLR + TTO.

Many studies evaluating outcomes after MPFL reconstruction and RTS rely on patient-reported measures of self-perceived function, including the International Knee Documentation Committee score, Tegner Activity Scale, Banff Patellofemoral Instability Instrument 2.0, and Kujala score.^2,3,11^ Although these instruments provide valuable insight into patients’ perceived knee function during recovery, they do not specifically assess the psychological component of recovery. Psychological factors are known to influence readiness to RTS in adolescents undergoing ACLR, but their role in recovery after MPFLR, with or without TTO, remains less well defined.^4^ Ryan et al^17^ found through a systematic review that fear of reinjury was a top reason for athletes not returning to sport after MPFLR, underscoring the need to further evaluate the psychological component of recovery in these patients. Hurley et al^6^ evaluated psychological readiness to RTS using a modified ACL-RSI questionnaire in a group of patients undergoing MPFLR with a mean age of 24 years, finding that patients with scores <56 were at greater risk for not returning to sports, with fear of reinjury being commonly cited as the reason for not returning. These results are similar to the cutoff value of 59.2 identified within the current study, supporting the value of this scoring threshold in predicting successful RTS. Given the established importance of psychological readiness in populations with anterior cruciate ligament injury, as well as emerging evidence that psychological and physical recovery may proceed at different rates after patellar stabilization surgery, it is important to measure and consider psychological readiness to create a more holistic understanding of recovery.^6^ Future research is needed to further examine the measurement properties of the ACL-RSI in this population and to refine cutoff scores for RTS across age- and sex-specific subgroups.

This study has several limitations, primarily related to its retrospective design, which may introduce selection and information biases. Our analysis was constrained by the data documented by providers regarding injury characteristics, sports participation, and patient outcomes. The use of retrospectively collected patient-reported outcome measures introduces additional limitations. Response bias may occur if patients with particularly favorable or poor outcomes were more likely to complete the surveys, and nonresponse bias limits our ability to assess outcomes in patients lost to follow-up. The timing of ACL-RSI collection may also influence responses, as patients’ perceptions of their outcomes can change over time, and data were not consistently collected at uniform postoperative intervals. Patient-reported outcome measures, however, remain a valuable tool for capturing the patient’s perspective on health, quality of life, and functional status after patellar stabilization surgery. Finally, unequal sample sizes across follow-up timepoints and between surgical groups limit the statistical power of between-group comparisons. Furthermore, the relatively small sample size of our MPFLR + TTO cohort (n = 27), and the inherent demographic and anatomic differences and increased baseline risk of patients indicated for TTO may limit the generalizability of our findings, and the interpretation of the impact of surgical procedure on the likelihood of RTS was not felt to be appropriate in the present study. Despite these limitations, our study provides important insights into factors influencing RTS after patellar stabilization surgery, as well as novel data on psychological benchmarks for assessing readiness to RTS in this population. Additional research is needed to confirm the validity of the ACL-RSI in this patient population and to support and improve its use in guiding clinicians’ assessment of psychological readiness for RTS.

Adolescent patients undergoing patellar stabilization surgery demonstrated RTS rates similar to those reported in predominantly adult cohorts in the existing literature. After adjustment for sex, our study identified that patients with ACL-RSI scores ≥59.2 were significantly more likely to achieve successful RTS. While further validation of this instrument is warranted in the PFI population, incorporation of psychological assessment tools, such as the ACL-RSI, into postoperative evaluations for patients undergoing patellar instability surgery may be beneficial to identify patients at risk for failure to RTS and to implement targeted interventions to help address the psychological barriers.
